# From research to practice: a model for clinical implementation of evidence-based outpatient interventions for eating disorders

**DOI:** 10.1186/s40337-021-00491-9

**Published:** 2021-11-12

**Authors:** Kristen E. Anderson, Sara G. Desai, Rodie Zalaznik, Natalia Zielinski, Katharine L. Loeb

**Affiliations:** Chicago Center for Evidence Based Treatment, 25 E Washington Street, Suite 1015, Chicago, IL 60602 USA

**Keywords:** Eating disorders, Family-based treatment, Private practice, Training, Multidisciplinary

## Abstract

**Background:**

A question frequently raised in the field is whether evidence-based interventions have adequate translational capacity for delivery in real-world settings where patients are presumed to be more complex, clinicians less specialized, and multidisciplinary teams less coordinated. The dual purpose of this article is to (a) outline a model for implementing evidence-driven, outpatient treatments for eating disorders in a non-academic clinical setting, and (b) report indicators of feasibility and quality of care.

**Main Body:**

Since our inception (2015), we have completed nearly 1000 phone intakes, with first-quarter 2021 data suggesting an increase in the context of COVID-19. Our caseload for the practice currently consists of approximately 200 active patients ranging from 6 to 66 years of age. While the center serves a transdiagnostic and trans-developmental eating disorder population, modal concerns for which we receive inquiries are Anorexia Nervosa and Avoidant Restrictive Food Intake Disorder, with the most common age range for prospective patients spanning childhood through late adolescence/emerging adulthood; correspondingly, the modal intervention employed is Family-based treatment. Our team for each case consists, at a minimum, of a primary internal therapist and a physician external to the center.

**Short Conclusion:**

We will describe our processes of recruiting, training and coordinating team members, of ensuring ongoing fidelity to evidence-based interventions, and of training the next generation of clinicians. Future research will focus on a formal assessment of patient outcomes, with comparison to benchmark outcomes from randomized controlled trials.

## Plain English summary

A question frequently raised in the eating disorders field is whether treatments that were developed and tested in research environments can achieve the same results in real-world clinical settings, where patients’ diagnoses are presumed to be more complex, clinicians less specialized, and multi-professional care teams less coordinated. The purpose of this article is to outline a model for implementing evidence-driven, outpatient treatments for eating disorders in non-academic clinical settings, specifically private practices and specialty programs. We describe the philosophy, infrastructure, training processes, personnel, and procedures utilized to optimize care delivery and to create accountability for both scientifically-adherent practice and positive patient outcomes. We also outline ways to be producers—not just consumers—of research in the private sector, and to train the next generation of scientifically-informed eating disorder specialists, all with the goal to bridge the research-practice divide.

## Introduction

A question frequently raised in the field is whether evidence-based interventions originating from highly specialized and controlled environments have adequate translational capacity for delivery in real-world settings, where patients are presumed to be more complex, clinicians less specialized, and multidisciplinary teams less coordinated. Moreover, the dissemination and implementation literature has identified several systems-level barriers including the extensive time commitment required for training and treatment delivery, the paucity of qualified trainers and resources, and a lack of support from practice administrators [1, 2]. However, recent studies examining cognitive behavioral therapy for eating disorders have demonstrated clinical outcomes similar to research outcomes [3–5], exemplifying that successful dissemination of other evidence-based interventions is plausible. In this article, we outline a model for implementing evidence-driven, outpatient treatments for eating disorders in a non-academic clinical setting (specifically private practices and specialty programs), and report indicators of feasibility and quality of care. We will describe our processes of developing a community-based practice including recruiting, training, and coordinating team members, ensuring ongoing fidelity to evidence-based interventions, and training the next generation of clinicians. The overarching purpose of this paper is to illustrate the replicability and dissemination potential of our model and to support community-based eating disorder therapists in developing or enhancing their practice paradigms. This aim resonates with ongoing efforts in the eating disorders field to bridge the research-practice divide via transparency, communication, and collaboration. Future research will focus on a formal assessment of patient outcomes, with comparison to benchmark outcomes from randomized controlled trials.

## Scope of the practice

The Chicago Center for Evidence Based Treatment (CCEBT) is an outpatient program serving children, adolescents, and adults with eating, feeding, and weight disorders, and other related and comorbid conditions. The primary goal and mission of CCEBT is to provide fidelity-driven treatments typically found in “ivory tower” academic settings in a community-based practice setting. We apply an evidence-based, multidisciplinary framework to our case conceptualization, assessment, treatment delivery, and clinical decision-making processes. From existing dissemination and implementation models [6], we primarily emphasize the training and supervision of clinicians, continuous review of quality indicators, and implementation support for evidence-based treatments in our administrative infrastructure. In addition to the clinical arm of our program, CCEBT offers training opportunities for future mental health professionals and conducts research in healthcare utilization of and intervention strategies for youth with Anorexia Nervosa (AN), Avoidant Restrictive Food Intake Disorder (ARFID), and transdiagnostic eating disorder presentations. Our trainees help us fulfill our commitment to maximizing access to care for all by providing affordable specialty treatment to individuals and families from the Chicago-area community. CCEBT currently has a grant-related academic affiliation with the Division of Adolescent Medicine at the University of Illinois at Chicago.

We maintain an active caseload of approximately 200 patients. While the center serves a transdiagnostic and trans-developmental eating disorder population (including Bulimia Nervosa (BN), Binge Eating Disorder (BED), and Other Specified Feeding or Eating Disorder (OSFED), our modal referrals are for children or adolescents with suspected AN or ARFID; correspondingly, and the modal intervention employed is family-based treatment (FBT) [7]. Our team for each case consists, at a minimum, of a primary therapist and a physician external to the center. We will describe our processes of developing a community-based practice including recruiting, training, and coordinating team members, ensuring ongoing fidelity to evidence-based interventions, and training the next generation of clinicians. Future research will focus on a formal assessment of patient outcomes, with comparison to benchmark outcomes from randomized controlled trials.

**Fig. 1:**
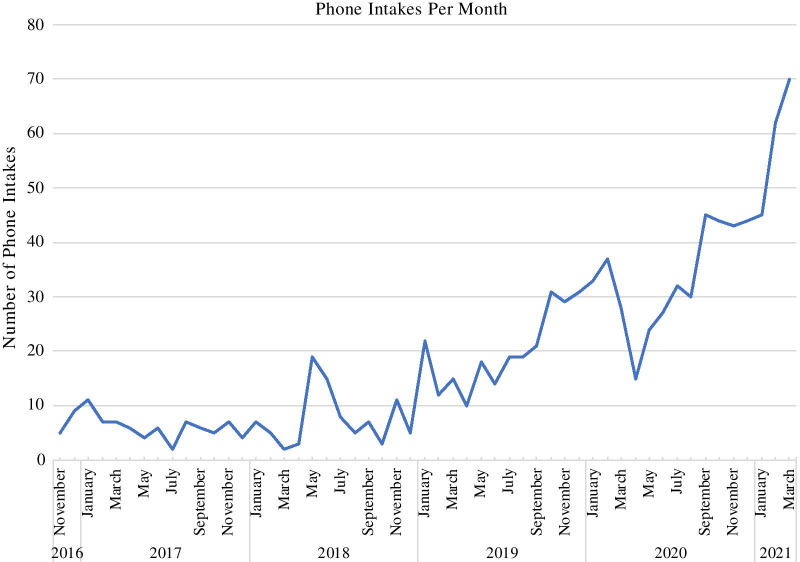
Volume of phone intakes per month and year

Since our inception in 2015, we have completed nearly 1000 phone intakes (Fig. 1). The majority of our referrals have come from professionals (physicians: 34%; therapists: 23%; school services and counselors: 10%; psychiatrists: 6%; dieticians: 1%) who are concerned that an adolescent patient has a restrictive eating disorder. Of those inquiring about treatment at CCEBT and completing an initial phone intake, half scheduled an in-person assessment (453 or 48%), or are on our waiting list (17 or 2%). The major reasons an assessment was not scheduled were insurance or cost concerns (26%), no availability with the desired therapist and/or a decision to seek treatment elsewhere (24%), or the patient instead proceeding to a higher level of care (9%). A quarter (25%) of individuals did not respond to any further contact from our clinical coordinator or program manager.

## Background

### The founders

The founders of CCEBT, Kristen Anderson, a Licensed Clinical Social Worker (LCSW) and Certified Eating Disorder Specialist Supervisor (CEDS-S), and Sara Desai, also a LCSW were previously members of a clinical and research team focused on eating disorders at an urban academic medical center. Anderson is a Certified FBT Provider and Supervisor, as well as a Faculty Member of the Training Institute for Child and Adolescent Eating Disorders (TICAED). She is also a CEDS-S through the International Association of Eating Disorder Professionals (IAEDP). Desai is a Certified FBT Provider. In their academic positions, they contributed to the development and execution of clinical research trials, learning successful strategies for flexible adherence to research-supported interventions in the management of complex clinical cases. They grew professionally into leadership roles, focusing on staff development and team cohesion. Importantly, they observed a smooth integration of science and practice at the medical center, an ethos they embraced and brought to the formation of CCEBT, where the team utilizes best practice guidelines derived from randomized controlled trials, while also factoring in individual comorbidities, diversity-based factors, and systems-level considerations.

### The idea

Personal life transitions brought an awareness of the importance and interdependence between the quality of life of clinicians and the quality of care for clients. This idea inspired the founders to create CCEBT. The partnership allowed for support on both the operational and logistics sides of the business as well as refining the clinical delivery model. To foster a strong sense of community at CCEBT and to protect fidelity to evidence-based protocols, we intentionally hired a team of therapists that shared the founders’ values, per recommendations from the literature [8, 9]. Awareness of the “leaky pipeline” for women in academia [8] motivated us to forge an environment that prioritized clinicians’ autonomy regarding intensity and flexibility of work schedules, allowing for the prioritization of quality of life outside the workplace without compromising the provision of high-quality, highly specialized evidence-based treatment to our patients.

## Creating the infrastructures: nuts and bolts

The considerations for trans-developmental eating disorder community-based providers include attention to the physical office space, the unexpected resource needs, and even the positioning of furniture. While the practice has grown substantially over the last five years, we have continued to ensure that the office space and logistics reflect the needs of patients and their families, and to provide a welcoming and comfortable space for our team. Specifically, we found that for clinicians to maintain adherence to the FBT [7] and Enhanced Cognitive Behaviour Therapy (CBT-E) [10] models, the following infrastructure must be in place: offices large enough to accommodate a therapist plus at least a moderate-sized family; a table and chairs for in-vivo family meals and space in which to conduct such a session; and a high-quality scale plus stadiometer. Over time, we have reconfigured space plans and purchased additional scales to ensure that each office suite is flexible to meet the needs of individuals and families. It is important that clinicians consider the placement of scales to allow for a private place to record weight, while being out of the patient’s sight during sessions so as to not create undue distraction. Additionally, considerations for an inclusive and safe space to accommodate diverse populations include physical accessibility, accessibility of office location to public transportation, and availability of gender-neutral bathrooms.

To ensure that therapists have access to key clinical materials, the practice provides a library of treatment manuals and workbooks for clinicians, as well as access to reliable and valid measures to track patient outcomes. Such questionnaires can be directly accessed, completed, and submitted through the patient portal of the Health Insurance Portability and Accountability Act (HIPAA)-compliant electronic health record system utilized by the center. This online, user-friendly option permits regular collection of psychometrically-sound measures of inter-session and end-of-treatment symptom change, a critical feature of evidence-based practice. The electronic health record also structures therapists’ documentation of session content, including treatment strategies applied and patients’ progress toward their goals and objectives. In both regards, the electronic health record system provides a framework for accountability and attention to behaviorally-specific indicators of patient response to intervention, and thereby supports our practice values while also serving as a passive monitor of protocol fidelity. Similarly, we capitalize on the center’s webpage less as a means to advertise CCEBT and more as a method of serving the public through education about eating disorders, scientifically-informed treatments, and resources such as articles, books, podcasts, and other reference materials.

## Making a meaningful connection from the start: the phone intake process

We developed our intake process to introduce prospective patients to the core philosophies of an evidence-based practice for eating disorders. Specifically, from the point of first contact, patients and/or their parents are exposed to fundamental tenets of treatment including the importance of: (a) an accurate diagnosis to inform treatment planning (e.g., for a family calling for treatment of a young child whose presentation might meet criteria for AN or ARFID), (b) favoring an actuarial clinical decision-making process over a subjective one (i.e., we start with the treatment for which the data show the strongest scientific likelihood of a good outcome, we do not assume that an intervention will not be appropriate for a particular patient unless research shows clear evidence of relevant moderator effects or there are clear clinical contraindications, etc.), and (c) the idea that eating disorders pose existing or potentially emerging medical and psychiatric crises that are urgent in nature (e.g., if a prospective patient is losing weight and our practice has no openings, we refer them to another provider rather than placing them on a waiting list; we require our active patients to see a physician for medical evaluation, clearance for outpatient treatment, and ongoing monitoring; we return intake calls within 24 h if possible; and we empower parents of children and adolescents to proceed with securing treatment even when met with extreme resistance). Thus, while there is an administrative component to the intake process, it is also an important clinical and psycho-educational intervention. Our program manager is a clinical social worker who previously worked as one of our therapists. She is familiar with the population we serve, the different treatment modalities that our practice offers, and the research behind them. This is particularly helpful when explaining FBT to families who are new to exploring the different types of treatment for eating disorders, or families who have heard or read about FBT and are interested but have questions or concerns. The program manager is also familiar with the specific certifications, competencies, and sub-specialties of each of our therapists, which is helpful when determining which clinician will be the right fit for each patient and their family.

## Managing patient risk (and therapist risk too)

In our experience, there are several considerations to maintain adherence to evidence-based treatment while supporting our patients and their families to maintain safety on an outpatient basis. These include managing medical and psychiatric risks, as well as evaluating progress and comorbidities to determine if additional interventions or a higher level of care may be warranted. Clear communication between members of the treatment team is of utmost importance in evaluating and communicating progress to the patient and parents [11]. Due to the risks associated with eating disorders such as bradycardia, electrolyte imbalance, dehydration, and suicidality, among other concerns, it is imperative that a clinician feels well supported in order to appropriately support the patient and their family, in turn. CCEBT’s founders have established a policy to be available on an on-call basis for clinicians. Practice therapists are in regular contact with co-treating physicians and we utilize the local Emergency Room to triage medical and psychiatric risk.

In an effort to maintain adherence to the FBT model of care, a majority of our clients remain in treatment on an outpatient basis. In the instance that a higher level of care is indicated, the team typically intensifies treatment within the assigned modality. This may include more frequent visits, utilizing the adaptive protocol for FBT [12], or referring families to Multi-Family Therapy (MFT) [13]. CCEBT has established relationships with medical providers across the Chicagoland area for patients who require hospitalization. During medical and psychiatric hospitalizations, we advocate for parents to be involved in the renourishment process and included in the treatment plan for their young person. While we typically recommend waiting until or after Phase 2 of FBT to incorporate indicated adjunctive psychological interventions when the patient is more nourished, in some circumstances we have utilized treatments such as Dialectical Behavior Therapy (DBT) [14, 15] or Exposure and Response Prevention (ERP) [16] alongside FBT to manage comorbidities. This requires close contact and coordination of care between the therapist teams.

An additional consideration of operating our program has been managing therapist burnout. When constructing the management of referrals and caseload distribution, the practice owners were intentional about maintaining reasonable caseloads and offering employees autonomy regarding their schedules. We were cognizant of the standard academic or group model which often requires a high number of cases per week. In order to manage therapist burden, we have recommended to our team that they take on less than a standard caseload, particularly when delivering FBT. This allows for time to consult with outside providers and to regularly utilize supervision, thereby minimizing burnout and maintaining positive patient outcomes [9, 17, 18]. When developing our fee structure, we accounted for a session duration of 50–60 min, and the intensity of communication required to provide quality care. In effort to make treatment more accessible, we developed a process to provide low-cost treatment through a student clinic, and a reduced fee schedule.

## Teamwork makes the dream work

With the aim of maintaining comprehensive care for our clients, we collaborate with healthcare providers in psychiatry, pediatrics, adolescent medicine, gastroenterology, speech therapy, and occupational therapy. Establishing our network of care providers has been a combination of luck and persistence. Initially, we developed a collaborative agreement with an adolescent medicine physician at an area hospital. This ongoing relationship and collaboration has been integral in the delivery of adherent manualized treatment. Due to this physician’s expertise and commitment to the treatment of eating disorders, we are able to treat children, adolescents, and young adults that may have otherwise required higher levels of care. This relationship allows us to treat patients just as we would on a team in academia, with regular communication and management of cases. As our practice has grown we have continued to build a network of providers specialized in eating disorders. This network is strengthened by frequent communication and the opportunity to present at case conferences and other educational seminars. This collaborative approach provides a unified team for our patients and their families [19]. In our model, therapists regularly speak to the adolescent medicine physicians by phone, and we have adopted a “clinic rounds” meeting once a month with each physician team to discuss cases in more depth. We also work very closely with several adult and child psychiatrists as well as other therapists who specialize in DBT, couples counseling and family therapy. The importance of these networks cannot be overstated as they allow the families we see to feel contained and supported throughout the treatment process. They also facilitate a process where the medical/mental health team is on the proverbial “same page”, which has been instrumental in delivering evidence-based treatments. Moreover, the establishment of relationships with other clinicians and physicians is of no cost to the practice, but immense benefit.

Quality control of service delivery across clinicians has been an ongoing process ensuring continuous fidelity to evidence-based treatment protocols, a challenge documented amongst FBT providers the further they progress into treatment [20, 21]. Notably, only about two-thirds of FBT therapists report using manual recommendations, and even those therapists report omitting key parts of treatment such as weighing the patient at every session [21]. Sources of therapist drift include patient and therapist reluctance to use more challenging, head-on approaches, as well as therapists’ intimidation of and low competency with a manualized protocol [22–24]. In effort to reduce therapist drift, we ensure that all of our clinicians are trained within the evidence-based models they utilize in treatment. Clinicians who offer FBT are either currently certified or are pursuing certification through the TICAED. Additionally, our clinicians have completed the Centre for Research on Eating Disorders at Oxford (CREDO) training for CBT-E, have received training in FBT-ARFID, and have received training and supervision in Cognitive Behavioral Treatment for ARFID (CBT-AR). Our clinicians often attend continuing education trainings as a group. We offer both group consultation and one-on-one supervision weekly. As a team, we integrate expert external consultation to ensure that therapists have support with complex cases. The variety and flexibility of support for therapists protects their autonomy and sense of control, while also ensuring fidelity by reducing therapist anxiety and raising confidence in their mastery of the protocol [9, 17].

## Growth and expansion: future focused

CCEBT has steadily grown throughout the last five years. What started with a single office in Chicago, is now three office locations serving Chicago and the suburbs. The co-founders continue to dedicate time to the recruitment of clinicians that share the founders’ philosophies, values, and standards. The initial team of two founders/clinicians has grown to twelve. Currently, our team is comprised of 10 therapists, 1 student trainee, and a master’s level program manager. This team includes 4 director-level therapists, including the co-founders, a director of research and training, and a director of adult services.

The founders of CCEBT are also dedicated to continuing the research component outside of academia. In conjunction with the University of Illinois at Chicago, we have been awarded a Health Care Services Corporation Affordability Cures Grant to (a) analyze national benchmark healthcare utilization for adolescent eating disorders data, and (b) test whether implementation of MFT for AN alters clinical trajectories relative to these benchmark data. This grant further solidifies CCEBT’s affiliation and collaboration with the adolescent medicine division at the University of Illinois at Chicago.

We are optimistic that the dissemination of evidence-based treatments will continue to increase as clinicians in the field continue to develop programs across the world to deliver these much-needed interventions. In an effort to publish these community-based treatment data, we have partnered with other practices nationally with the goal of disseminating data related to indicators of fidelity to treatment as well as clinical outcomes. These research goals reflect our practice values by continuing to hold CCEBT to as many of the same standards inherent in randomized controlled trials as possible by conducting formal, aggregate assessments of patient outcomes, with comparison to benchmark outcomes from key studies utilizing the same interventions. We hope to expand research efforts to include staging treatment interventions, including the additions of DBT and other evidence-based treatments to FBT as well as understanding how we can best support clients for whom our standard treatments have proved inadequate.

The training and supervision of the next generation of therapists are of paramount importance. The social work internship and psychology externship at CCEBT are designed to (a) provide an advanced, specialized treatment-delivery experience to trainees while (b) increasing broader, transdiagnostic knowledge and competencies in: the common phenomenology and mechanisms of psychopathology; evidence-based and ethical clinical decision-making; operating within an interdisciplinary framework; crisis management; diversity issues; and professionalism. The practicum is focused on graduate students interested in one or more of the following areas: eating disorders, anxiety disorders, families, or children and adolescents. Our efforts to reduce direct patient care hours to prioritize supervision and training are a feature of our program. When the student therapist joins our team, they are required to attend intensive pre-requisite workshops and didactics (e.g., in FBT and CBT) prior to treating cases. Supervision is conducted in a combination of group and individual formats, with opportunities to also participate in professional-level peer consultation meetings. The secondary goal of the student clinic is to provide expert-supervised low-cost/no-cost treatment to the community. This allows CCEBT to provide clinical treatment to underserved populations.

## Conclusion

In the dissemination of this practice model, solo or smaller practices should not be discouraged. CCEBT grew to twelve clinicians from an initial two by utilizing resources including no-cost options and by building a network of collaborations. Start-up costs can be lowered by relying on training sessions offered by professional organizations and speciality institutes, many of which simultaneously satisfy continuing education required for continued professional licensure. The emphasis on building strong relationships with fellow clinicians and physicians cannot be overstated and serves a two-fold purpose of satisfying the requirements of delivering FBT, and building a continuous system of support for therapists.

Our goal for creating the practice was to bridge the research/practice divide and increase access to care outside of academic medical centers. We hope that we have provided the readers with enough practical information and insight to feel that this is a feasible and highly rewarding format in which to provide clinical treatment. We believe that evidence-based interventions do indeed have adequate translational capacity for delivery in real-world settings like ours given the implementation of multidisciplinary care, attention to and execution of reliable practice protocols, and allowing an abundance of opportunities for training and consultation that are paramount for preserving the wellbeing of clinicians and patients alike.

## Data Availability

Not applicable.

## Abbreviations

AN: Anorexia Nervosa
BED: Binge Eating Disorder
BN: Bulimia Nervosa
ARFID: Avoidant Restrictive Food Intake Disorder
CBT-E: Cognitive Behavioral Therapy
CCEBT: Chicago Center for Evidence Based Treatment
CEDS-S: Certified Eating Disorder Specialist Supervisor
CREDO: Centre for Research on Eating Disorders at Oxford
DBT: Dialectical Behavior Therapy
ERP: Exposure and Response Prevention
FBT: Family Based Treatment
HIPAA: Health Insurance Portability and Accountability Act
IAEDP: International Association of Eating Disorder Professionals
IOP: Intensive Outpatient Program
LCSW: Licensed Clinical Social Worker
MFT: Multi-Family Therapy

## References

[1] Couturier JL, Kimber MS (2015). Dissemination and implementation of manualized family-based treatment: a systematic review. Eat Disord.

[2] Fairburn CG, Wilson GT (2013). The dissemination and implementation of psychological treatments: problems and solutions. Int J Eat Disord.

[3] Byrne SM, Fursland A, Allen KL, Watson H (2011). The effectiveness of enhanced cognitive behavioural therapy for eating disorders: an open trial. Behav Res Ther.

[4] Knott S, Woodward D, Hoefkens A, Limbert C (2014). Cognitive behaviour therapy for bulimia nervosa and eating disorders not otherwise specified: translation from randomized controlled trial to a clinical setting. Behav Cogn Psychother.

[5] Turner H, Marshall E, Stopa L, Waller G (2015). Cognitive-behavioural therapy for outpatients with eating disorders: effectiveness for a transdiagnostic group in a routine clinical setting. Behav Res Ther.

[6] Southam-Gerow MA, McLeod BD (2013). Advances in applying treatment integrity research for dissemination and implementation science: introduction to special issue. Clin Psychol Sci Pract.

[7] Le Grange D, Lock J (2013). Treatment manual for anorexia nervosa a family-based approach (2nd edition).

[8] Ysseldyk R, Greenaway KH, Hassinger E, Zutrauen S, Lintz J, Bhatia MP (2019). A leak in the academic pipeline: identity and health among postdoctoral women. Front Psychol.

[9] Kim JJ, Brookman-Frazee L, Gellatly R, Stadnick N, Barnett ML, Lau AS (2018). Predictors of burnout among community therapists in the sustainment phase of a system-driven implementation of multiple evidence-based practices in children’s mental health. Prof Psychol Res Pract.

[10] Fairburn CG (2008). Cognitive behavior therapy and eating disorders.

[11] Couturier J, Kimber M, Barwick M, Woodford T, McVey G, Findlay S (2018). Themes arising during implementation consultation with teams applying family-based treatment: a qualitative study. J Eat Disord.

[12] Lock J, Le Grange D, Agras WS (2015). Can adaptive treatment improve outcomes in family-based therapy for adolescents with anorexia nervosa? Feasibility and treatment effects of a multi-site treatment study. Behav Res Ther.

[13] Eisler I, Simic M, Hodsoll J, Asen E, Berelowitz M, Connan F (2016). A pragmatic randomised multi-centre trial of multifamily and single family therapy for adolescent anorexia nervosa. BMC Psychiatry.

[14] Linehan MM (1993). Cognitive-behavioral treatment of borderline personality disorder (diagnosis and treatment of mental disorders).

[15] Linehan MM (2015). DBT skills training handouts and worksheets.

[16] Foa EB, Yadin E, Lichner TK (2012). Exposure and response (ritual) prevention for obsessive-compulsive disorder: therapist guide.

[17] Aarons GA, Fettes DL, Flores LE, Sommerfeld DH (2009). Evidence-based practice implementation and staff emotional exhaustion in children's services. Behav Res Ther.

[18] Glisson C, Hemmelgarn A (1998). The effects of organizational climate and interorganizational coordination on the quality and outcomes of children’s service systems. Child Abuse Negl.

[19] Loeb KL, Sanders L. Physician-therapist collaboration in family-based treatment for anorexia nervosa: What each provider wants the other to know. In: Innovations in family therapy for eating disorders: novel treatment developments, patient insights, and the role of careers. New York: Routledge, Taylor & Francis Group; 2016.

[20] Dimitropoulos G, Freeman VE, Allemang B, Couturier J, McVey G, Lock J (2015). Family-based treatment with transition age youth with anorexia nervosa: a qualitative summary of application in clinical practice. J Eatng Disord..

[21] Kosmerly S, Waller G, Robinson AL (2014). Clinician adherence to guidelines in the delivery of family-based therapy for eating disorders. Int J Eat Disord.

[22] Couturier J, Kimber M, Jack S, Niccols A, Van Blyderveen S, McVey G (2012). Understanding the uptake of family-based treatment for adolescents with anorexia nervosa: therapist perspectives. Int J Eat Disord.

[23] Waller G (2009). Evidence-based treatment and therapist drift. Behav Res Ther.

[24] Waller G (2016). Treatment protocols for eating disorders: clinicians’ attitudes, concerns, adherence and difficulties delivering evidence-based psychological interventions. Curr Psychiatry Rep.

